# Exercise Addiction in Practitioners of Endurance Sports: A Literature Review

**DOI:** 10.3389/fpsyg.2018.01484

**Published:** 2018-08-17

**Authors:** Abel Nogueira, Olga Molinero, Alfonso Salguero, Sara Márquez

**Affiliations:** Department of Physical Education and Sports, Institute of Biomedicine, University of León, León, Spain

**Keywords:** exercise addiction, exercise dependence, behavior, running, endurance, marathon, athletes

## Abstract

Practice of endurance sports/activities has gained most devotees over recent decades, thanks to its capacity to maintain and improve health. However, their characteristics and accessibility have facilitated the emergence of addictive behaviors. Excessive practicing can lead to adverse physical and psychological effects seriously prejudicial to health, to the extent that individuals are unable to control this behavior. Recognizing that exercise addiction is still a controversial concept, the aim of the present review is to analyze the state of research into addictive exercise behaviors, specifically centering on running and endurance sports. To this end, a search covering article written in English and dated from 2010 onward was carried out in the Pubmed, Scopus, Web of Science and EBSCOhost databases. Of a total of 2,675 articles in the four databases, 25 were eligible for the final review. The studies reviewed confirmed that excessive practice could encourage the appearance of addictive behaviors and health problems. Most research has reported no age or sex differences in exercise dependence among endurance sport practitioners; however, obsessive passion and dedication to sports activities may be predictors for addiction to exercise. Owing to terminological confusion and the variety of tools used to measure addiction, figures for prevalence differ widely among studies, with values quoted ranging from 3 to 42%. Furthermore, it is clear that there are problems in delimiting, defining and diagnosing this sort of behavior, which has led to addiction to exercise not yet being considered a behavioral disorder.

## Introduction

The concept of addiction originally referred to an excessive and out-of-control consumption of psychotropic substances, but increasingly applies to a group of syndromes known as “behavioral addictions” (Pinna et al., 2015). Of all these, the DSM-5 (American Psychiatric Association, 2013) holds gambling addiction, in which the focus of the addiction is presented by a specific behavior, to be the sole disorder that fulfills the characteristics common to substance addictions. The WHO's ICD-11 Working Group on Obsessive-Compulsive Related Disorders considers a category of impulse control disorders, which also includes compulsive sexual behavior (Grant and Chamberlain, 2016). Although addiction to exercise is not recognized as such, different authors consider that it could fall into the category of behavioral addictions. Hausenblas and Downs (2002b) operationalized the specific addiction to exercise as a multidimensional maladaptive pattern that leads to a disability or a clinically significant affliction, manifested by the presence of at least three of the seven criteria included in the DSM-IV (American Psychiatric Association, 1994). More recently, Starcevic (2016) divided potentially addictive behaviors into two categories, including addiction to exercise in the second grouping. As happens in other situations, people addicted to exercise go through periods in which they are incapable of controlling their behavior because of the pleasure that undertaking this activity brings them, regardless of any negative consequences (Berczik et al., 2011; Sellman, 2016). Moreover, social acceptance of sport as a behavior strongly positive for health (Forrest et al., 2016), even for those people with a high risk of becoming addicts (Lichtenstein et al., 2017), makes it more difficult to understand that exercise can be an addiction. In any case, although exercise dependence has crossed the boundary of a disorder, exercise addiction is still a controversial concept (Starcevic and Khazaal, 2017), an operational definition of behavioral addictions with a number of exclusion criteria (which cover high-level sports) has been recently proposed (Kardefelt-Whinter et al., 2017), and there is a need for well-founded discussion in this area (Kräplin, 2017; Starcevic, 2016). An Open Science Framework (OSF) has been recently created, supporting further development to build a conceptualization of behavioral addictions in a transparent, collaborative and iterative manner (Billieux et al., 2017).

In 1976, Glasser began to refer to the concept of positive addiction to exercise, in order to differentiate the positive effects of this behavior from the negative consequences associated with other addictive conduct, fundamentally behaviors linked to the consumption of substances (Glasser, 1976). Shortly afterwards, Morgan (1979) suggested that exercise might have possible negative outcomes. From that point onwards, a wide range of terms have been used to describe and refer to this behavior, leading to a context of conceptual confusion (Macfarlane et al., 2016). Among the most frequent expressions are obligatory, abusive, compulsive or excessive exercise, and exercise dependence (Cockerill and Riddington, 1996; Farrell and Thompson, 1998; Davis, 2000; Hausenblas and Downs, 2002a,b; Dalle Grave et al., 2008; Fairburn, 2008; Freimuth et al., 2011; Meyer and Taranis, 2011). Nonetheless, despite the large number of terms used, there is general agreement that when a regularly exercising person loses control over her or his exercise behavior, that is, when the behavior increases in frequency and converts in a necessity, it can result in detrimental effects. In this situation, the affected individual is unable to properly pay attention or concentrate on other daily activities (Mónok et al., 2012; Cook et al., 2013, 2014).

Exercise dependence may be defined as a behavioral process in which individuals obtain pleasure or achieve relief from difficulties, but which causes negative consequences for them and for their immediate circle of family and friends (Bircher et al., 2017). Its manifestation or appearance is not sudden or abrupt. Rather, it is a process characterized by the presence of six symptoms common to all addictions: salience (or prominence in the addict's life), mood modification (the “high”), tolerance, withdrawal (involving symptoms), conflict (with the personal circle) and relapse (Brown, 1993; Griffiths, 2005; Sussman and Sussman, 2011). Addiction to exercise can be classified as primary, in which exercise is a direct mediator for psychological anguish (Szabo, 2010), or secondary (replacement), in which individuals use exercise as a means of maintaining or attaining a state of fitness and a desired body shape. This means that the latter often appears in conjunction with other types of psychological dysfunctions such as anorexia, bulimia, or both (Bamber et al., 2000; Blaydon et al., 2002).

Of all the types of sport studied (Ogden et al., 1997; Szabo and Griffiths, 2007; Lindwall and Palmeira, 2009; Sicilia and González-Cutre, 2011; Parastatidou et al., 2012; Lichtenstein et al., 2014), endurance sports are those showing the greatest risk of addiction. In 1984, Sachs and Pargman introduced by first time the concept of addiction to exercise, under the name of running addiction, to describe the source of a set of withdrawal symptoms that surface during periods of running deprivation Sachs and Pargman (1984). These authors tried to confirm the study executed by Morgan (1979), who provided examples in which runners continued to run despite adverse circumstances. Masters et al. (1993), used the term super-adherence to refer to the fact that while approximately one half of the people who start a physical activity program drop out in the first 6 months, those who decide to prepare a marathon rarely drop out, becoming this activity one important part of their lives which leads to what is known as negative addiction to running (NAR). Results obtained from studies carried out on endurance sport practitioners, especially those taking part in marathons (Allegre et al., 2007; Smith et al., 2010; Modoio et al., 2011; Salas et al., 2013), have shown that they have higher probabilities to be in risk to suffer addiction than other sports folk.

Different models have attempted to explain the origin and continuance of addiction to exercise (Szabo et al., 2016). There are psychological models, like the Affect Regulation Hypothesis (Tomkins, 1968), the Cognitive Appraisal Hypothesis (Szabo, 1995), the Four-Phase Model of Exercise Addiction (Freimuth et al., 2011), the Biopsychosocial Model (McNamara and McCabe, 2012), the Interactional Model for Exercise Addiction (Egorov and Szabo, 2013) and the Interactive Model (Berczik et al., 2014), which is closely related to the PACE (Pragmatics, Attraction, Communication, Expectation) model worked out for addictions in general (Sussman et al., 2011). Physiological models, such as the Sympathetic Arousal Hypothesis (Thompson and Blanton, 1987), the Interleukin-6 Model (Hamer and Karageorghis, 2007), the Catecholamines Hypothesis (Cousineau et al., 1977), and the role of biochemical markers (creatine kinase and lactate dehydrogenase) in relation to emotions (Antunes et al., 2016), have been also proposed.

However, the most influential hypothesis relates to the phenomenon known as “runner's high.” This is a sensation of euphoria that has been attributed to the central effects of endorphins and other endogenous opioids, seen as responsible for whether or not dependences or addictive behaviors appear (Dishman and O'Connor, 2009; Dubreucq et al., 2010; Raichlen et al., 2012; Kraemer et al., 2013). Many practitioners of physical exercise, especially runners, commonly experience these neurobiological rewards, during and after distance running (Antunes et al., 2016). Endorphins produced by the body are converted in their own opiate-like peptides, which can cause dependence (and consequently may be the route of withdrawal symptoms (Szabo et al., 2013b). Because beta-endorphin is secreted and modify its levels during vigorous exercise (Dishman and O'Connor, 2009), different studies have examined the effects of exercise intensity on endogenous opioid production during cycling, running on a treadmill and running marathons (Szabo et al., 2013b). Sensations experienced have been described as a state of sheer joy, euphoria, inner harmony, limitless energy, feelings of wellbeing and a reduced perception of pain (Raichlen et al., 2012). Such emotions and sensations very similar to those described by drug addicts and people addicted to other types of substances (Kanarek et al., 2009). The connection between beta-endorphins and runner's high is a suitable explanation for exercise addiction in endurance activities, although more empirical support is still required (Szabo et al., 2013b).

On the basis of the data noted here a systematic literature review on addiction to exercise was carried out in order to establish the present state of knowledge in endurance sports and especially long- and middle-distance running. The works by Bircher et al. (2017), Kempf et al. (2017), and Liao et al. (2015) were taken as a benchmark, with the aim of learning which terms were most often used, and the prevalence, causes and risks of suffering this behavioral pattern.

## Method

The search string was built in collaboration by the four authors of the study, who constructed the inclusion criteria, searched and evaluated the relevant literature. Information was obtained from the databases Pubmed, Scopus, Web of Science and EBSCOhost using combinations by Boolean logic of the following keywords: “exercise addiction,” “exercise dependence,” “exercise abuse,” “excessive exercise,” “compulsive exercise,” “obligatory exercise,” “exercise and passion,” “behavioral addictions and exercise,” “running,” “runners,” “athletes,” “half marathon,” “marathon,” “long distance,” “endurance,” “triathlon,” and “trail running.” The search was limited to those articles written in English and published between January 2010 and December 2017.

**Figure 1:**
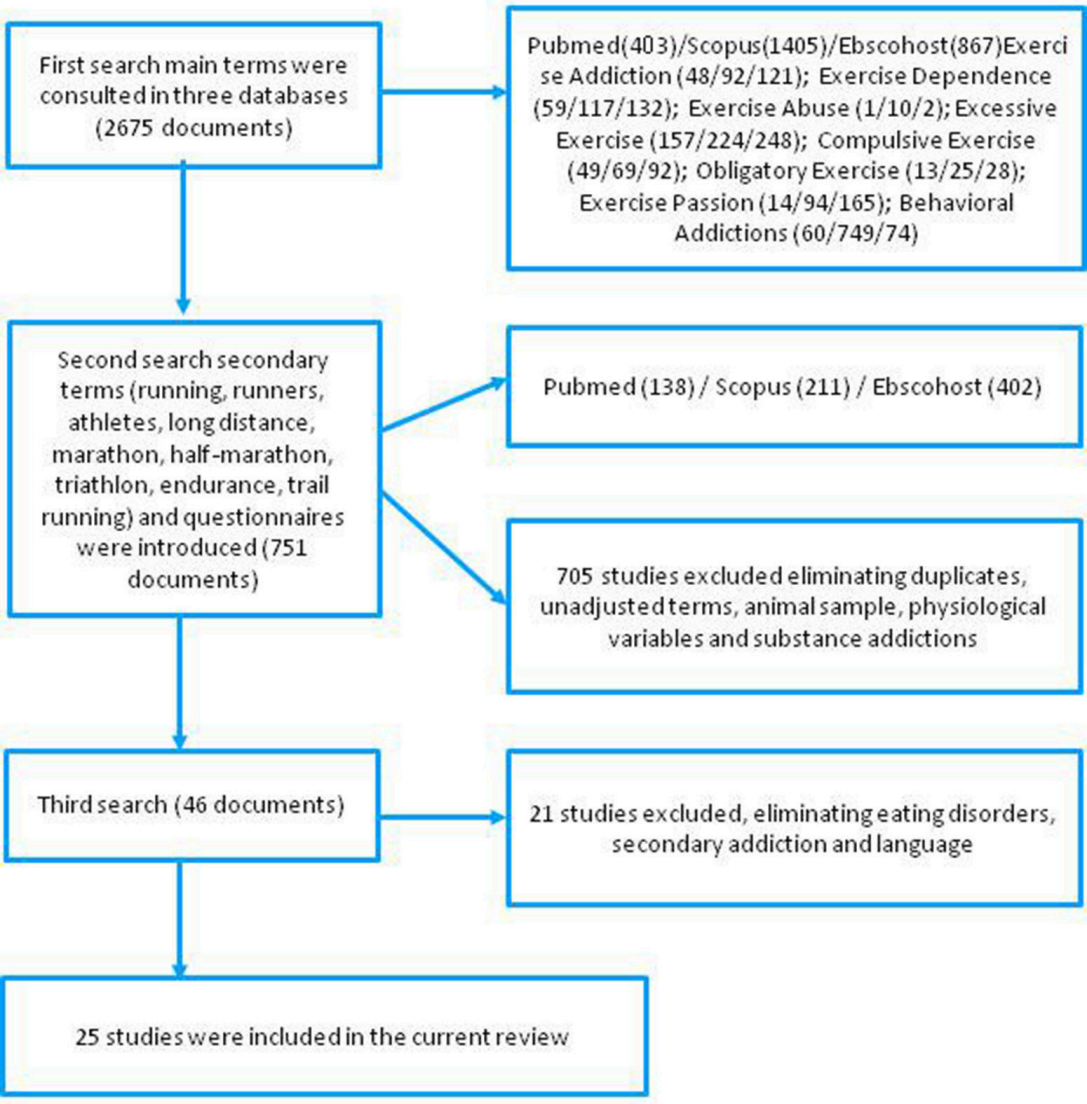
Quality assessment of included studies.

The first search sweep used all the combinations of the keywords quoted above, and yielded 2,675 potentially relevant articles. In a second phase secondary terms were added (see Figure 1), reducing the number of studies to 751. Of these, 701 were excluded because they used animal samples, concentrated on the analysis of physiological variables (hypertension, arrhythmia, cardiovascular damage) or addressed substance addiction. In a final phase, a further 21 articles were ruled out because they were duplicates, had as their aim the study of secondary addiction or dependency and feeding disorders, or both.

In the end, 25 articles were eligible for the final review, covering studies with samples of endurance athletes and sports that included foot races. Moreover, they included all those studies using as tools the Commitment to Running Scale (CR, Carmack and Martens, 1979), the Running Addiction Scale (RAS, Chapman and De Castro, 1990) and the Motivations of Marathoners Scales (MOMS, Masters et al., 1993). The eligibility of these articles was independently assessed and confirmed by all four authors via an in-depth critical full-text review. A schematic representation of the search procedure is shown in Figure 1.

## Results

Table 1 summarizes the general characteristics of the 25 include studies. Half were published during the last 3 years, among them two bibliographic reviews and two descriptive studies. The term most widely used by researchers was addiction (eleven articles). Among the factors chosen to check on possible relationships with addiction, the most prominent were motivation (five articles), training, well-being and anxiety (three articles each), and gender (two articles).

**Table 1:** Summary of studies selected in the review (2010–2017).

**Study**	***N***	**Sex**	**Age (mean)**	**Sport**	**Objectives**	**Instruments**	**Conclusions**
Ertl et al., 2017	322	Female	19.9	Recreational, elite athletes	Examine predictors of exercise addiction.	-Exercise addiction inventory-short form (Terry et al., 2004)	-Body shame and self-esteem could be predictors of exercise addiction
Martin et al., 2017	20	Male, female	40.9	Running	Examine behavioral and neural measures of impulsivity in endurance runners	-Exercise addiction inventory (Terry et al., 2004)	-Endurance runners not only report addictive symptoms related to exercise, but also demonstrate addictive-like behaviors
Mayolas-Pi et al., 2017	859	Male, female		Cycling, inactive	Determine the relationship between the risk of exercise addiction (REA)	-Spanish version exercise addiction inventory (Sicilia et al., 2013)	-REA in amateur endurance cycling is not significantly influenced by extrinsic factors (age, sex, training, etc.)
Rivera Rodríguez et al., 2017	30	Male, female	32.9	Running	Experimentally assess the effect on central nervous activation of cognitive strategies of association and dissociation while running		-Tasks of association and dissociation did not cause fatigue among participants and did not impair. -A positive effect on central nervous activation was observed.
Schiphof-Godart and Hettinga, 2017				Running	Examine the influence of passion in sport on athletes' short term and long-term decision-making and exercise behavior		-Obsessive passion may affect athlete well-being and performance on the long run
Antunes et al., 2016	18	Male		Running	Identify the possible association between biochemical markers of exercise addiction and affective parameters	-Portuguese version of the negative addiction scale (Rosa et al., 2003) -Exercise dependence scale Hausenblas and Downs (2002a)	-2-week withdrawal exercise resulted in an increase of negative mood in exercise addiction. -Exercise addiction showed low levels of anandamide
Buning and Walker, 2016	408	Male, female			Explore participant motivations to compete in two different mass participant sport events	-Motivations of marathoners scales (Masters et al., 1993)	-Motivations more significant are: health, weight concern, personal goal achievement, affiliation, psychological coping, life meaning, and self-esteem
de la Vega et al., 2016	313	Male, female	28.6	Regular exercisers	Determine the link between exercise addiction and harmonious passion, obsessive passion, and dedication to sports, in the context of athletic levels	-The re-validated Spanish version (Sicilia et al., 2013) of the 6-item EAI (Terry et al., 2004) -Spanish adapted version of Passion Scale (SPS; Chamarro et al., 2015)	-Athletes could interpret exercise addiction screening-items differently from non-athletes. -Athletes in team sports report greater passion and dedication than those practicing individual sports.
Lucidi et al., 2016	669	Male, female	42.1	Running (Marathon)	Examine the relation between regulatory modes, locomotion and assessment, and stress	-The Italian version of the Passion Scale (Marsh et al., 2013)	-Locomotion positively predicted harmonious passion, which in turn reduced athletes' experience of stress -Locomotion positively predicted obsessive passion, which in turn enhanced athletes' experience of stress
Zach et al., 2015	346	Male, female	41.9	Running	Test and expand the Motivation of Marathoners Scale model	-Motivations of marathoners scales (Masters et al., 1993)	-The new MOMS model obtained better psychometric soundness
Zarauz-Sancho et al., 2016	1795	Male, female	*M* = 39.0 *F* = 37.9	Running (Trail)	Find out which predictive relationships would be introduced by motivation, commitment to run, negative addiction to run and pre-competition anxiety	-Spanish version of Motivations of Marathoners Scales-34 (Ruiz-Juan and Zarauz, 2011a) -Spanish version of the Commitment to Running Scale-11 (Ruiz-Juan and Zarauz, 2011b) -Spanish version of the Running Addiction Scale-8 (Zarauz Sancho and Ruiz-Juan, 2011)	-As in similar studies, the orientation to ego like the task, have been similar and moderate -Cultural differences between nationalities, regarding to commitment to run -The principal predictive variables were major motivation of overcoming personal-competition goals and a big part of self-confidence
Cook et al., 2015	1766	Female	37.0	Running	Investigate the unique and interactive effect of the exercise identity and social physique anxiety with the exercise dependence	-Exercise dependence scale (Hausenblas and Downs, 2002a)	-Exercise identity may be a factor in the development and maintenance of exercise dependence
Hanson et al., 2015	865			Running (Marathon, Ultramarathon)	Compare the motives of half, full and ultramarathoners and to create a profile of male ultramarathoners	-Motivations of marathoners scales (Masters et al., 1993)	-Ultramathoners were less motivated by health orientation and weight concern, but more motivated by affiliation and life meaning -Women were more motivated to run to control weight
Szabo et al., 2015, Review					Do a brief analytical review to highlight and disentangle research dilemmas in the field of exercise addiction		-There is no consistency in describing addictive exercise behavior -It is needed for consistent terminology -Self-report instruments only provide a risk score, and due to inconsistent interpretations related to the nature of the studied sample
Rundio et al., 2014	167	Male, female	42.2	Triathlon, cycling	Discover which are the events attract athletes and know the motives of athletes to participate in cause-related or non-cause-related sport events	-Motivations of marathoners scales (Masters et al., 1993)	-The five motivations more important: general health orientation, personal goal achievement, weight concern, self-esteem, and affiliation motivations -The motivations for participate in cause-related sport events: self-esteem, recognition/approval, personal goal achievement and competition reasons -The motivations for participate in non-cause-related sport events: weight concern
Schüler et al., 2014	29	Male, female	47.5	Ultraendurance	Study how the implicit achievement and affiliation motives interact with the need for competence and the need for social relatedness satisfaction, respectively, to predict flow experience and well-being in extreme endurance athletes	-German version of the positive and negative affect schedule (Krohne et al., 1996)	-The satisfaction of the basic needs for competence and social related- ness alone did not predict the flow experience and facets of well-being in extreme endurance sports equally for everybody -Interaction between basic need satisfaction and the respective motive dispositions -Positive consequences of basic need satisfaction in sports
Youngman and Simpson, 2014	1285	Male, female		Triathlon	Investigate the risk of exercise addiction for triathletes	-Exercise addiction inventory (EAI; Terry et al., 2004)	-No significant association between the risk for exercise addiction and the number of years of participating -More number of weekly training hours, the risk for exercise addiction is higher
Weinstein and Weinstein, 2014, Review					Summarize phenomenology of exercise addiction with emphasis on physiological and neuropharmacological mechanisms responsible for its rewarding and addictive properties		-Regular exercise taken into excess may have adverse physiological and psychological consequences
Karr et al., 2013	2421	Male, female	37.7	Running (marathon, half-marathon)	Analyze the association between exercise identity and obligatory exercise	-Obligatory exercise questionnaire (Pasman and Thompson, 1988; Thompson and Pasman, 1991)	-Women who participate in athletic events, maintain high exercise identity, and internalize the athletic-ideal body shape may be vulnerable to developing obligatory exercise cognitions and behaviors
Szabo et al., 2013a	242		27.5	Non-sport University Athletes Ultramarathon	Examine the influence of gender, social context (team or individual sport), and level of athletic training on symptoms of exercise addiction	-The Spanish version of the 6-item exercise addiction inventory (Terry et al., 2004)	-Gender, level of athletic training and social context of the training, affect exercise addiction -The volume of exercise did not emerge as an index of susceptibility to exercise addiction
Lane and Wilson, 2011	34	Male, female		Running	To research relationships between trait emotional intelligence and emotional state changes over the course of an ultra-endurance foot race	-Brunel mood scale (Terry et al., 2003)	-Runners high in self-report trait emotional intelligence, reported higher pleasant emotions than runners low in trait emotional intelligence
Modoio et al., 2011	300	Male, female			To check if there are differences between male and female athletes' scores on measures of negative addiction symptoms, quality of life, mood and sleep	-Negative addiction scale (Hailey and Bailey, 1982)	-No differences were seen in the development of negative addiction exercise symptoms in males and females -No changes in the quality of life and mood of these athletes
Zarauz Sancho and Ruiz-Juan, 2011	*N*_1_ = 174, *N*_2_ = 975	Male, female	*M*_1_ = 41.29, *M*_2_ = 39.67	Running	Present the first preliminary psychometric data of the Spanish version of RAS and to analyze the internal structure of the instrument	-Running addiction scale (Chapman and De Castro, 1990)	-The Spanish version of the RAS showed acceptable levels of internal consistency, temporal stability, inter-item correlations, total scale score and construct validity
Smith et al., 2010	184	Male, female	28.1	Running	Study the differences in exercise dependence and social physique anxiety between competitive and non-competitive runners	-Exercise dependence scale-21 (Hausenblas and Downs, 2002a) -Running addiction scale (Chapman and De Castro, 1990)	-Competitive runners are more likely to exhibit symptoms of exercise dependence and lower exhibit more social physique anxiety
Shipway and Holloway, 2010	25	Male, female		Running	Explore the implications for sport and leisure policy of how distance running could positively contribute to healthy living and physical well-being	-Interviews and observation	-Distance running provides a complex mix of both positive and negative experiences and provides one potential route to a healthy lifestyle

The average number of subjects used in investigations was 558. Of the pieces of work, seventeen had samples including both genders, three chose women only for their sample and just one had an all-male sample. The most extensively studied type of sport was long distance running (28%), followed by marathon (20%), triathlon (8%) and cycling (8%). It is worth highlighting the fact that one of the articles compared runners at risk displaying symptoms of addiction with others not showing these symptoms.

Finally, in respect of the tools most often used, six of the studies employed the Exercise Addiction Inventory (Terry et al., 2004), followed by the Motivations of Marathoners Scale (MOMS) (Masters et al., 1993) and the Exercise Dependence Scale (Hausenblas and Downs, 2002a), which were used in three. Only one of the studies made use of a combination of three questionnaires: CR (Commitment to Running), RAS (Running Addiction Scale) and MOMS.

## Discussion

Regular physical exercise is an activity with a major capacity to maintain and improve physical and mental health (Shipway and Holloway, 2010; Mayolas-Pi et al., 2017). Nonetheless, in the light of the results of research, excessive practicing may cause serious health problems, giving rise to the appearance of addictive behaviors (Weinstein and Weinstein, 2014). The articles analyzed demonstrate that the problems already identified in respect of the definition, diagnosis and etiology of addiction to exercise (Szabo et al., 2015; Kardefelt-Whinter et al., 2017) are still present. They are in part responsible for this behavior not yet being seen as a mental disorder, therefore not included in the latest edition of the DSM, despite its proven relationship with mental disturbances (Weinstein et al., 2015), eating disorders (Blaydon and Lindner, 2002; Bratland-Sanda et al., 2010) and other behavioral addictions (Villella et al., 2011; Müller et al., 2015), or the availability of tools possessing adequate construct validity (Zarauz Sancho and Ruiz-Juan, 2011; Youngman and Simpson, 2014).

Many models have attempted to explain this behavior. However, the idea underlying most of them is that exercise has the power to constitute a positive reinforcement, besides its ability to act as a stress-reduction strategy. In turn, it has been noted that the genes which control a liking for drugs are also responsible for naturally gratifying behaviors like exercise. It is at this point that running and endurance sports have a differentiating role, being seen as types with an antidepressant capacity which have the potential to reduce psychological distress through pleasure induction by activating endogenous opiates (Weinstein and Weinstein, 2014). This is borne out by the number of endurance athletes who state that they started practicing this sport as a way of beating some other addiction, or as a means of reducing stress. Nevertheless, in many instances their strong dedication and the immediate gratification received turns them into exercise addicts (Lee et al., 2017). For their part, Antunes et al. (2016) demonstrated that deprivation of exercise for 2 weeks caused a decline in feelings of well-being, with detection of low levels of anandamide endocannabinoids and an increase in the levels of β-endorphin.

The results of research on participants in endurance sports give evidence for a relationship between exercise commitment and exercise dependence (Lu et al., 2012). This is due to the heavy demands (Murray et al., 2013) and considerable number of hours and sessions given over to training (Salas et al., 2013; Szabo et al., 2013a). Examples are the triathletes studied by Youngman and Simpson (2014), or the marathon runners investigated by Karr et al. (2013) and Zarauz-Sancho et al. (2016), these authors finding a positive correlation between the total hours spent training and the risk of addiction to exercise.

Other variables taken into account are sex and age. With regard to the latter, most research has detected no significant differences by age (Modoio et al., 2011; Zarauz Sancho and Ruiz-Juan, 2011; Szabo et al., 2013b; Youngman and Simpson, 2014; Mayolas-Pi et al., 2017). Although an early study on a small group of marathon racers found that women scored significantly higher in exercise dependence than men (Pierce et al., 1997), there are no other reports of sex differences in exercise addiction among runners, in spite of differences in motives for participation; women usually run more because of a preoccupation with controlling weight and body image, whilst men do so because of the impact of a social and competitive nature that practicing this sport provides (Modoio et al., 2011; Hanson et al., 2015; Ertl et al., 2017). For their part, Buning and Walker (2016), Rundio et al. (2014), and Schüler et al. (2014) demonstrated that runners' motivation differs according to the characteristics of the event, attracting them as a function of the degree to which their essential motives are fulfilled and their basic needs met.

In recent years, research has attempted to seek out the relationship between addiction to exercise and other factors such as passion (Vallerand, 2012), considering this to be a useful tool for appropriate training and for supervising the well-being of athletes (de la Vega et al., 2016; Lucidi et al., 2016; Schiphof-Godart and Hettinga, 2017), and Kovacsik et al. (in press) have shown a relationship between the risk for exercise addiction, exercise intensity and passion. Lane and Wilson (2011) found that runners underwent significant changes in their emotions during runs, besides demonstrating that emotional intelligence correlates with pleasant feelings in the course of such events. More recently, Rivera Rodríguez et al. (2017) have described long-distance running is beneficial when it comes to completing tasks that require keeping cognitive effort at a high level of vigilance, selective attention, decision-making, cognitive control, self-regulation and motor behaviors.

Finally, owing to terminological confusion and the variety of tools used to measure exercise dependence, figures for the prevalence of this behavior differ widely among studies, with values quoted ranging from 3 to 42% (Smith et al., 2010; Lejoyeux et al., 2012; Mónok et al., 2012; Szabo et al., 2015; Lichtenstein and Jensen, 2016). Whatever the figure, these are still worrying data far from concealing the emergence of a new reality that is coming closer and closer to turning into a serious problem for health in present-day society. Indeed, a recent piece of research undertaken by Martin et al. (2017) has highlighted the fact that people practicing endurance sports continue despite being injured and in addition have high scores on the Inventory of Addiction to Exercise. The practitioners of endurance sports studied pressed on in spite of the negative consequences brought about by not running in the best physical condition, because the recompense they derive is greater than any reward from not doing so. Competitive runners show a greater number of symptoms of addiction when compared to non-competitive, regardless of their sex (Smith et al., 2010). In any case, effort in future research in this field should be focused on conceptualizing, delimiting, unifying and studying the role of various different factors in the development of addiction to exercise (Cook et al., 2015). The aim should be an attempt to guide or divert sports activities in the direction of health (Smith et al., 2010).

## Author contributions

All authors listed have made a substantial, direct and intellectual contribution to the work, and approved it for publication.

### Conflict of interest statement

The authors declare that the research was conducted in the absence of any commercial or financial relationships that could be construed as a potential conflict of interest.
